# Clinical features and outcomes of breakthrough vitreous hemorrhage secondary to polypoidal choroidal vasculopathy

**DOI:** 10.1371/journal.pone.0279778

**Published:** 2022-12-30

**Authors:** Meng-Syuan Li, Chui-Lien Tsen

**Affiliations:** 1 Division of Ophthalmology, Pingtung Veterans General Hospital, Pingtung, Taiwan, R.O.C; 2 Department of Ophthalmology, Kaohsiung Veterans General Hospital, Kaohsiung, Taiwan, R.O.C; 3 Department of Optometry, Shu-Zen Junior College of Medicine and Management, Kaohsiung, Taiwan, R.O.C; Bascom Palmer Eye Institute, UNITED STATES

## Abstract

Polypoidal choroidal vasculopathy (PCV) with hemorrhagic complications is at higher risk for breakthrough vitreous hemorrhage (VH). This study aimed to evaluate the clinical features and outcomes of breakthrough VH secondary to PCV. Data of patients receiving pars plana vitrectomy for breakthrough VH secondary to PCV (VH group) were evaluated retrospectively and compared statistically to data of age and sex-matched PCV patients without breakthrough VH (control group). Among PCV patients, 36 eyes with breakthrough VH and 62 eyes without VH were included. Compared with baseline, best corrected visual acuity (BCVA) was worse in the VH group (*P* < 0.001), and improved postoperatively (P < 0.001). Percentages of pigmented epithelial detachment (PED), hemorrhagic PED, massive subretinal hemorrhage, hemorrhagic retinal detachment (RD), and hemorrhagic choroidal detachment (CD) (P = 0.007) were higher in the VH group (*P* < 0.001). Incidence of choroidal vascular hyperpermeability (*P* < 0.001), massive subretinal hemorrhage (*P* = 0.001), hemorrhagic retinal detachment (*P* = 0.001) and hemorrhagic type PCV (*P* = 0.001) was higher in patients with pachychoroid PCV, while fibrovascular type had lower incidence (*P* < 0.001). Better initial BCVA (*P* < 0.001), higher frequency of anti-VEGF treatment (*P* = 0.009), and previous photodynamic therapy (*P* = 0.017) showed better visual outcomes. Breakthrough VH risk is higher in PCV patients with massive subretinal hemorrhage, hemorrhagic PED and hemorrhagic RD. BCVA and hemorrhagic complications improve significantly postoperatively. Higher frequency of anti-VEGF treatment and previous photodynamic therapy are associated with better visual prognosis in PCV patients with breakthrough VH.

## Introduction

Polypoidal choroidal vasculopathy (PCV) is a vision-threatening disease characterized by polyp-like dilations of choroidal vessels. PCV may cause pigmented epithelial detachment (PED), serous retinal detachment (RD), massive subretinal hemorrhage and extensive macular atrophy. For many decades, PCV has been described as a subtype of neovascular age-related macular degeneration (nAMD). In 1990, Yannuzzi et al. introduced a new term–idiopathic polypoidal choroidal vasculopathy (IPCV)—to describe their finding of an exudative form of macular disorder that did not fall into the category of AMD [1]. In 2013, Warrow et al. proposed a new term, “pachychoroid,” to describe a spectrum of disease characterized by thickened choroid with choroidal congestion and choroidal vascular hyperpermeability (CVH) [2]. Greater understanding of PCV in recent years has led ophthalmologists to consider that some PCV cases had a different pathogenesis than nAMD that may originate from pachychoroid-driven choroidal neovascularization rather than subretinal neovasculization. Furthermore, the epidemiology, risk factors, clinical presentation, and prognosis are also quite different between nAMD and PCV. Compared with nAMD, patients diagnosed with PCV are younger, female-predominant, and pigmented races are preferentially affected [3]. On indocyanine green (ICG) angiography, PCV is characterized by branched, dilated vessels with single or multiple polyp-like lesions at the level of choroid, which show dye leakage in the late phase.

Before photodynamic therapy (PDT) and anti-vascular endothelial growth factor (anti-VEGF) treatments were introduced, most ophthalmologists treated PCV conservatively unless persistent or progressive exudative change threatened patients’ vision [3]. Thermal laser treatment and diode laser photocoagulation were used but were limited in treating extra-foveal PCV due to their destructive nature [4, 5]. Anti-VEGF and PDT treatments have become popular treatments for PCV due to their effectiveness and safety, even in cases with subfoveal PCV. However, even while under treatment, patients with PCV may still encounter massive subretinal hemorrhage or breakthrough vitreous hemorrhage (VH), which may lead to sudden, severe, and possibly irreversible vision loss. This study aimed to identify factors influencing the final visual outcomes of PCV between patients with or without breakthrough VH.

## Methods

### Study design and sample

This observational study retrospectively reviewed medical records of patients who were diagnosed with breakthrough VH secondary to PCV during treatment between December 2009 and May 2021 at Kaohsiung Veterans General Hospital, a tertiary referral center in southern Taiwan. A group of age and sex-matched patients who were diagnosed with PCV during the same period but without breakthrough VH were included as the control group. All patients underwent a comprehensive eye examination, including best corrected visual acuity (BCVA), slit-lamp biomicroscopy, dilated fundus examination, color fundus photography, optical coherence tomography (OCT), and ICG angiography. The diagnosis of PCV was made by experienced retinal specialists after reviewing results of the examination described above, according to the diagnostic criteria reported in the EVEREST study [6].

### Ethical considerations

This observational case-series study adhered to the tenets of the Declaration of Helsinki and was approved by the Institutional Review Board and ethics committee of Kaohsiung Veterans General Hospital (approval number: 21-CT1-30(201223-1). Patients’ informed consent was waived due to the retrospective design of this study, which analyzed pre-existing patient data.

### Data collection and main variables

Patients’ demographic data (age and sex), and clinical data, including systemic diseases, lens status, treatment methods for PCV, features of PCV on fundus exam, ICG angiography and optical coherence tomography (OCT), were collected from patients’ medical records. Massive subretinal hemorrhage was defined as thick submacular bleeding that extended past the equator in at least two quadrants. Pachychoroid PCV was diagnosed according to the features of reduced tessellation on fundus photography, dilated choroidal vessels on OCT and ICG angiography, CVH on ICG angiography and increased choroidal thickness on enhanced depth imaging OCT. Patients with VH that was not secondary to PCV, and patients with concomitant retinal disorders (e.g., macular pucker, macular hole, rhegmatogenous/tractional retinal detachment, diabetic macular edema) or retinal/choroidal vasculopathy (e.g., diabetic retinopathy, retinal vein/artery occlusion, retinal vasculitis, uveitis) were excluded. Patients with media opacities such as compromised lens or corneal clarity were also excluded.

### Statistical analysis

All statistical analyses were performed using IBM SPSS Statistics software (IBM SPSS Statistics for Windows, Version 22.0. Released 2013. IBM Corp. Armonk, NY, USA). For statistical analysis, the BCVA was converted from the Snellen decimal chart to logarithm of the minimum angle of resolution (LogMAR) values. Visual acuity recorded counting fingers was converted to 1.98 and hand motion was converted to 2.28 in LogMAR based on the Freiburg Visual Acuity Test for quantifying visual acuity of patients with very low vision, as previously described [7]. Descriptive statistics are expressed as mean and standard deviation. Means for normally distributed variables were compared using the student’s *t* test. Variables without normal distribution were compared using the nonparametric Mann-Whitney *U* test. Multiple linear regression was used to evaluate factors associated with BCVA at final visits. Chi-square test was used to analyze categorical variables. A two-tailed P value of less than 0.05 was regarded as statistically significant.

## Results

The data of 179 patients diagnosed with PCV and VH and 56 PCV patients without breakthrough VH as control cases were reviewed. After excluding 155 patients who met the exclusion criteria, 98 eyes of 80 patients were included for statistical analysis, including 36 eyes of 36 patients of the study group and 62 eyes of 44 patients of the control group. The mean follow-up period was 23.61 months. The baseline characteristics of these 98 eyes are summarized in Table 1. The mean age was 71.8 ± 10.6 years. Among the 98 eyes, 62 eyes belonged to male patients and 36 eyes belonged to female patients. Sixty-seven eyes were phakic (68.4%). Compared with the baseline status, the best corrected visual acuity (BCVA) was worse in the VH group than the non-VH group (LogMAR, 2.01 ± 0.77 vs. 0.73 ± 0.61, respectively; *P* < 0.001). In the VH group, percentages of PED (100 % vs. 56.5%, respectively; *P* < 0.001), hemorrhagic PED (66.7 % vs. 0%, respectively; *P* < 0.001), massive subretinal hemorrhage (72.2 % vs. 1.6%, respectively; *P* < 0.001), hemorrhagic RD (55.6 % vs. 0%, respectively; *P* < 0.001), and hemorrhagic choroidal detachment (CD) (11.1 % vs. 0%, respectively; *P* = 0.007) were higher than in the non-VH group. No significant differences were found between the VH and control group in age, sex, underlying systemic disease, using anti-coagulants, previous PDT, and pachychoroid status.

**Table 1:** Baseline demographic and clinical characteristics of patients with polypoidal choroidal vasculopathy.

	PCV (n = 80, Eyes = 98)			
Number of Patients and Eyes	General Data	PCV with Breakthrough VH	PCV without Breakthrough VH	*P*
		(n = 36, Eyes = 36, 36.7%)	(n = 44, Eyes = 62, 63.3%)	
**Age, years, mean** ± **SD**	71.8 ± 10.6	72.9 ± 12.4	71.2 ± 9.5	0.478
**Sex (male), n (%)**	62 (63.3)	21 (58.3)	41 (66.1)	0.440
**Hypertension, n (%)**	46 (46.9)	14 (38.9)	32 (51.6)	0.224
**Diabetes mellitus, n (%)**	22 (22.4)	7 (19.4)	15 (24.2)	0.587
**Use of anticoagulants, n (%)**	5 (5.1)	3 (8.3)	2 (3.2)	0.268
**BCVA, LogMAR, mean** ± **SD**	1.20 ± 0.91	2.01 ± 0.77	0.73 ± 0.61	<0.001*
**Previous PDT, n (%)**	9 (9.2)	4 (11.1)	5 (8.1)	0.615
**Previous pneumatic displacement, n (%)**	9 (9.2)	7 (19.4)	2 (3.2)	0.007*
**Frequency of anti-VEGF, time/month, mean** ± **SD**	0.58 ± 0.87	0.70 ± 1.36	0.51 ± 0.33	0.164
**CVH, n (%)**	61 (62.2)	24 (66.7)	37 (59.7)	0.491
**Cluster type, n (%)**	21 (21.4)	11 (30.6)	10 (16.1)	0.093
**Pachychoroid, n (%)**	66 (67.3)	24 (66.7)	42 (67.7)	0.913
**Presence of PED, n (%)**	71 (72.4)	36 (100)	35 (56.5)	<0.001*
**Type of PED**				
Fibrovascular, n (%)	38 (38.8)	18 (50.0)	20 (32.3)	0.082
Serous, n (%)	33 (33.7)	16 (44.4)	17 (27.4)	0.086
Hemorrhagic, n (%)	24 (24.5)	24 (66.7)	0 (0)	<0.001*
**Massive subretinal hemorrhage**	27 (27.6)	26 (72.2)	1 (1.6)	<0.001*
**Hemorrhagic RD**	20 (20.4)	20 (55.6)	0 (0)	<0.001*
**Hemorrhagic CD**	4 (4.1)	4 (11.1)	0 (0)	0.007*
**Extensive macular fibrosis**	13 (13.3)	6 (16.7)	7 (11.3)	0.449

*Represents statistical significance

PCV, polypoidal choroidal vasculopathy; SD, standard deviation; VH, vitreous hemorrhage; BCVA, best corrected visual acuity; LogMAR, logarithm of the minimum angle of resolution; PDT, photodynamic therapy; anti-VEGF, anti-vascular endothelial growth factor; CVH, choroidal vascular hyperpermeability; PED, pigmented epithelial detachment; RD, retinal detachment; CD, choroidal detachment

Tables 2 and 3 show subgroup analysis of the VH group. BCVA of the VH group improved after surgery (LogMAR, 2.01 ± 0.77 vs. 1.38 ± 0.83, respectively; *P* < 0.001). The frequency of intravitreal injections (IVI) of anti-VEGF decreased after breakthrough VH compared to baseline values (time/month, 0.70 ± 1.36 vs. 0.24 ± 0.25, respectively; *P* = 0.009). At the end of follow-up, no significant changes were shown between fibrovascular and serous PED compared with their initial status. Hemorrhagic PED (66.7% vs. 36.1%, respectively; *P* = 0.001), massive subretinal hemorrhage (72.2% vs. 16.7%, respectively; *P* < 0.001), and hemorrhagic RD (55.6% vs. 13.9%, respectively; *P* < 0.001) decreased compared to baseline values. The percentage of extensive macular fibrosis increased significantly at the end of follow-up compared with the initial status (16.7% vs. 41.7%, respectively; *P* = 0.004).

**Table 2:** Comparison of patients with breakthrough vitreous hemorrhage before and after treatment.

	Initial status	Final status	*P*
**BCVA, LogMAR, mean** ± **SD**	2.01 ± 0.77	1.38 ± 0.83	<0.001*
**Frequency of anti-VEGF, time/month, mean** ± **SD**	0.70 ± 1.36 (Pre-OP)	0.24 ± 0.25 (Post-OP)	0.009*
**Type of PED**			
Fibrovascular	18 (50)	19 (52.8)	1.000
Serous	16 (44.4)	11 (30.6)	0.063
Hemorrhagic	24 (66.7)	13 (36.1)	0.001*
**Massive subretinal hemorrhage, n (%)**	26 (72.2)	6 (16.7)	<0.001*
**Hemorrhagic RD, n (%)**	20 (55.6)	5 (13.9)	<0.001*
**Hemorrhagic CD, n (%)**	4 (11.1)	2 (5.6)	0.500
**Extensive macular fibrosis, n (%)**	6 (16.7)	15 (41.7)	0.004*

*Represents statistical significance

BCVA, best corrected visual acuity; LogMAR, logarithm of the minimum angle of resolution; SD, standard deviation; anti-VEGF, anti-vascular endothelial growth factor; OP, operation, PED, pigmented epithelial detachment; RD, retinal detachment; CD, choroidal detachment

**Table 3:** Difference in pachychoroid status of polypoidal choroidal vasculopathy with breakthrough vitreous hemorrhage.

	PCV with breakthrough VH			
Number of patients and eyes	Overall	Pachychoroid PCV	Nonpachychoroid PCV	*P*
	(n = 36)	(n = 24, 66.7%)	(n = 12, 33.3%)	
**Age, years, mean** ± **SD**	72.9 ± 12.4	68.7±11.9	81.3 ± 8.59	0.003*
**Sex (male), n (%)**	21 (58.3)	12 (50)	9 (75)	0.151
**Hypertension, n (%)**	14 (38.9)	7 (29.2)	7 (58.3)	0.091
**Diabetes mellitus, n (%)**	7 (19.4)	3 (12.5)	4(33.3)	0.137
**Use of anticoagulants, n (%)**	3 (8.3)	1 (4.2)	2 (16.7)	0.201
**Lens status (phakic), n (%)**	20 (55.6)	17 (70.8)	3 (25.0)	0.009*
**CVH, n (%)**	24 (66.7)	23 (95.8)	1 (8.3)	<0.001*
**Cluster type, n (%)**	11 (30.6)	9 (37.5)	2 (16.7)	0.201
**Previous anti-VEGF, n (%)**	34 (94.4)	23 (95.8)	11 (91.7)	0.607
**Previous PDT, n (%)**	4 (11.1)	3 (12.5)	1 (8.3)	0.708
**Previous pneumatic displacement, n (%)**	7 (29.2)	7 (29.2)	0 (0)	0.037*
**Surgical factors**				
Gas tamponade, n (%)	8 (22.2)	8 (33.3)	0 (0)	0.023*
S.O tamponade, n (%)	1 (2.8)	1 (4.2)	0 (0)	0.473
**Initial status**				
BCVA, LogMAR, mean ± SD	2.01±0.77	1.91±0.78	2.20±0.74	0.188
Frequency of anti-VEGF, time/month, mean ± SD	0.70±1.36	0.86±1.62	0.39±0.53	0.251
Presence of PED, n (%)	36 (100)	24 (100)	12 (100)	-
Type of PED				
Fibrovascular, n (%)	18 (50.0)	7 (29.2)	11 (91.9)	<0.001*
Serous, n (%)	16 (44.4)	13 (54.2)	3 (25.0)	0.097
Hemorrhagic, n (%)	24 (66.7)	22 (91.7)	2 (16.7)	<0.001*
Large sub-retinal hemorrhage	26 (72.2)	22 (91.7)	4 (33.3)	<0.001*
Hemorrhagic RD	20 (55.6)	18 (75.0)	2 (16.7)	0.001*
Hemorrhagic CD	4 (11.1)	3 (12.5)	1 (8.3)	0.708
Extensive macular atrophy	6 (16.7)	2 (8.3)	4 (33.3)	0.058
**Final status**				
BCVA, LogMAR, mean ± SD	1.38±0.83	1.19±0.77	1.75±0.84	0.052
Post-operative anti-VEGF, n (%)	26 (72.2)	18 (75.0)	8 (66.7)	0.599
Frequency of anti-VEGF, time/month, mean ± SD	0.24±0.25	0.27±0.27	0.18±0.22	0.379
Presence of PED, n (%)	36 (100)	24 (100)	12 (100)	-
Type of PED				
Fibrovascular, n (%)	19 (52.8)	10 (41.7)	9(75.0)	0.059
Serous, n (%)	11 (30.6)	9 (37.5)	2 (16.7)	0.201
Hemorrhagic, n (%)	13 (36.1)	11 (45.8)	2 (16.7)	0.086
Large sub-retinal hemorrhage, n (%)	6 (16.7)	4 (16.7)	2(16.7)	1.000
Hemorrhagic RD, n (%)	5 (13.9)	3(12.5)	2 (16.7)	0.733
Hemorrhagic CD, n (%)	2 (5.6)	1 (4.2)	1 (8.3)	0.607
Extensive macular fibrosis, n (%)	15 (41.7)	9 (37.5)	6 (50.0)	0.473
**Follow-up period, months, mean** ± **SD**	23.61 ± 22.27	26.79 ± 24.17	17.25 ± 4.918	0.231

*Represents statistical significance

PCV, polypoidal choroidal vasculopathy; SD, standard deviation; VH, vitreous hemorrhage; SD, standard deviation; CVH, choroidal vascular hyperpermeability; anti-VEGF, anti-vascular endothelial growth factor; PDT, photodynamic therapy; S.O, silicone oil; BCVA, best corrected visual acuity; LogMAR, logarithm of the minimum angle of resolution; PED, pigmented epithelial detachment; RD, retinal detachment; CD, choroidal detachment

Mean age of patients with pachychoroid PCV was younger than that in the non-pachychoroid group (68.7 ± 11.9 years vs. 81.3 ± 8.59 years, respectively; *P* = 0.003), and more patients in the pachychoroid group were phakic. Compared to non-pachychoroid patients, the pachychoroid group also had higher incidence of CVH (95.8% vs. 8.3%, respectively; *P* < 0.001), massive subretinal hemorrhage (91.7% vs. 33.3%, respectively; *P* < 0.001), hemorrhagic RD (75% vs. 16.7%, respectively; *P* = 0.001), and higher incidence of previous pneumatic displacement (29.2% vs. 0%, respectively; *P* < 0.037). Compared with the type of PED on OCT at initial status, patients with pachychoroid had higher incidence of hemorrhagic type (91.7% vs. 16.7%, respectively; *P* < 0.001) and less fibrovascular type (29.2% vs. 91.9%, respectively; *P* < 0.001). However, in contrast, no statistically significant differences were found in the type of PED between pachychoroid and nonpachychoroid groups at final status. The percentage of gas tamponade in the end of vitrectomy for VH was higher in the pachychoroid group (33.3% vs. 0%, respectively; *P* = 0.023).

The factors associated with visual prognosis are shown in Table 4. Patients who had better initial BCVA (*P* < 0.001), higher frequency of anti-VEGF treatment (*P* = 0.009), and previous photodynamic therapy (*P* = 0.017) were associated with better visual outcomes. The characteristics of PCV and the type of PED were not associated with visual outcomes.

**Table 4:** Factors associated with visual prognosis in polypoidal choroidal vasculopathy with breakthrough vitreous hemorrhage.

	N (%)/Mean ± SD	Final BCVA	Beta	*P* value
		(Mean LogMAR ± SD)		
**Number of eyes**	36 (100)	1.36±0.81		
**Initial BCVA, LogMAR**	2.01±0.77	-	0.566	<0.001*
**Previous treatment**				
Frequency of anti-VEGF, time/month	0.70 ± 1.36	-	-0.403	0.009*
PDT (+)	4 (11.1)	1.04 ± 0.43	-0.361	0.017*
PDT (-)	32 (88.9)	1.40 ± 0.84		
pneumatic displacement (+)	7 (29.2)	1.15 ± 0.81	-0.260	0.082
pneumatic displacement (-)	29 (70.8)	1.41 ± 0.81		
**Characteristics of PCV**				
Pachychoroid (+)	24 (66.7)	1.17 ± 0.73	-0.202	0.228
Pachychoroid (-)	12 (33.3)	1.75 ± 0.84		
CVH (+)	24 (66.7)	1.23 ± 0.81	0.273	0.369
CVH (-)	12 (33.3)	1.62 ± 0.76		
Cluster type PCV (+)	11 (30.6)	1.71 ± 0.60	0.147	0.317
Cluster type PCV (-)	25 (69.4)	1.21 ± 0.85		
**Type of PED**				
Fibrovascular (+)	18 (50.0)	1.63 ± 0.84	0.024	0.905
Fibrovascular (-)	18 (50.0)	1.09 ± 0.69		
Serous (+)	16 (44.4)	1.27 ± 0.91	0.031	0.827
Serous (-)	20 (65.6)	1.43 ± 0.73		
Hemorrhagic (+)	24 (66.7)	1.18 ± 0.80	0.048	0.853
Hemorrhagic (-)	12 (33.3)	1.70 ± 0.73		

*Represents statistical significance

SD, standard deviation; BCVA, best corrected visual acuity; LogMAR, logarithm of the minimum angle of resolution; anti-VEGF, anti-vascular endothelial growth factor; PDT, photodynamic therapy; PCV, polypoidal choroidal vasculopathy; CVH, choroidal vascular hyperpermeability; PED, pigmented epithelial detachment

## Discussion

Hemorrhagic complications, including subretinal hemorrhage, intraretinal hemorrhage, hemorrhagic RD or CD, and VH, are common in PCV patients, regardless of whether the patient is treatment naive or undergoing treatment. The present study demonstrates that the VH group had worse BCVA, higher incidence of PED, and hemorrhagic complications, including hemorrhagic PED, massive subretinal hemorrhage, hemorrhagic RD, and hemorrhagic CD. Preoperatively, 26 patients (72.2%) with massive subretinal hemorrhage were identified in VH group. In the remaining 10 patients, 1 had subretinal hemorrhage that did not meet the criteria of massive amount; subretinal hemorrhage in 3 patients and massive subretinal hemorrhage in 5 patients were identified intraoperatively; a small area of retinal hemorrhage was identified in 1 patient. The worse BCVA in the VH group may be associated with the higher incidence of hemorrhagic complications, of which the hemorrhage may involve the fovea, causing significant loss of vision.

In PCV, the active polypoidal lesions and abnormal vessel structures may induce leakage, leading to PED [8]. In the present study, the higher incidence of PED may be associated with higher activity of the polypoidal lesions and more fragile vessels in the VH group, increasing patients’ risk of hemorrhagic complications. Based on clinical observation and experience, VH usually occurs days or weeks after subretinal hemorrhage, and retinal breaks or holes are rarely found [9, 10]. In the present study, retinal breaks were only noted intraoperatively in 3 patients (2.8%) with hemorrhagic RD. In 2003, Lincoff et al. used an animal model and reported that thick subretinal hemorrhage causes necrosis of overlying retina and fragments of erythrocytes can cross an intact internal limiting membrane to cloud the vitreous [10]. In 2018, Shin et al. reported that the use of anticoagulants, large-diameter submacular hemorrhage, and PCV subtypes were risk factors for breakthrough VH after anti-VEGF injection in AMD [11]. These findings help to explain the phenomenon of breakthrough VH after massive subretinal hemorrhage without a retinal break, which is compatible with our finding of the positive association between breakthrough VH and hemorrhagic complications. However, the present study did not identify significant associations between the use of anti-coagulants and breakthrough VH in PCV patients. These results may be explained by the small population of anti-coagulant users included in our research.

In the VH group, BCVA and hemorrhagic complications improved significantly at the final status after pars plana vitrectomy and subsequent IVI of anti-VEGF. In the previous study, hemorrhagic RD, baseline central macular thickness, and baseline BCVA were reported as the factors associated with final BCVA in patients with breakthrough VH secondary to PCV [12]. In the present study, better initial BCVA, previous PDT, and higher frequency of anti-VEGF injections were identified and found to be associated with better final visual outcomes. These results suggest that adequate treatments before and after hemorrhage are still necessary to achieve better visual outcomes. However, hemorrhagic complications after PDT or anti-VEGF injection are not uncommon. The incidence of hemorrhagic complications such as subretinal hemorrhage or breakthrough VH are reported to range from 0% to 33% after PDT monotherapy and 0.6% to 23% after combination therapy of PDT and anti-VEGF injections [6, 13–23]. Hirami et al. suggested that laser irradiation during PDT may occlude choroidal vessels, and if reperfusion only occurs in the afferent vessels, the dammed blood flow may result in massive bleeding [14]. In the present study, we did not find significant associations between breakthrough VH and PDT but we found that the frequency of IVI of anti-VEGF decreased after breakthrough VH. In 2018, Baek et al. reported diminished activity of PCV after development of a large subretinal hemorrhage that was accessed by the reduced numbers of anti-VEGF injections. They also compared results of ICG angiography, which revealed that polypoidal lesions disappeared after hemorrhage in 70% eyes. Results of that study supported the postulation of polypoidal lesions regressing after rupture, resulting in subretinal hemorrhage and decreased disease activity, which translates into decreased frequency of anti-VEGF injection [24].

Untreated submacular hemorrhage had been reported to be associated with poor visual outcomes [8, 25, 26]. Experimental evidence also revealed that subretinal blood may cause retinal damage [10, 27–29]. Therefore, pneumatic displacement was introduced to remove blood from under the fovea [30]. However, acute vitreous hemorrhage after pneumatic displacement for submacular hemorrhage has still been reported [30–32]. The present study revealed a higher rate of previous pneumatic displacement for submacular hemorrhage in the VH group. In 7 patients in the VH group (19.4%) who received previous pneumatic displacement, and 5 patients developed VH in 2 weeks after pneumatic displacement (9 to 60 days). The incident rate of VH in the present study after pneumatic displacement was similar to that of previous reports [31, 33]. Patients with submacular hemorrhage should be well-informed of the risk of acute breakthrough VH and the possibility of vitrectomy for non-clearing VH before pneumatic displacement is performed.

As of now, the exact pathogenesis of PCV is still not well-established. Whether PCV shares the same pathogenesis as nAMD or is a distinct disease entity is still debatable. For several decades, studies have reported that PCV with pachychoroid is a different disease entity than that found in PCV patients with non-pachychorid PCV. Baek et al. compared the choroidal vascular characteristics of AMD, PCV, and central serous chorioretinopathy (CSCR), finding similarities between AMD and thin choroid PCV and between thick choroid PCV and CSCR [34]. Chang and Cheng analyzed the correlation between choroidal thickness and clinical features of 66 eyes with PCV and concluded that PCV patients could be subclassified into a pachychoroid group with younger age and more CSCR-like features, and a non-pachychoroid group with older age and more AMD-like features [35]. Zhao et al. reviewed 103 patients with the diagnosis of breakthrough VH secondary to PCV and found that patients were younger, all had hemorrhagic PED, and a higher prevalence of CVH [12]. In the present study, similar results included that PCV patients with pachychoroid were younger and had a higher rate of CVH. Furthermore, we identified more hemorrhagic complications, including hemorrhagic PED, hemorrhagic RD, and massive subretinal hemorrhage in the pachychoroid group at initial status. These hemorrhagic complications may result in relatively complicated surgery, which is reflected by the need of tamponade agents in the pachychoroid group. In contrast, the non-pachychoroid group presented with more fibrovascular PED, which was similar to the presentation of AMD, and fewer hemorrhagic complications, which made the surgery relatively uncomplicated.

The present study has several limitations, including a relatively small case number and the use of retrospective design, which limits inferences of causation and does not allow long-term follow-up. This study was also a single-center study with eight retinal specialists, which may induce bias due to different clinical experience, preferences of surgical methods, and different choices of anti-VEGF regimens. Besides, the control group has 62 eyes from 44 patients, which may give genetic contributions, leading to bias. Future prospective, randomized controlled study with larger sample size are needed to confirm findings of the present study.

In conclusion, PCV with hemorrhagic complications, including massive subretinal hemorrhage, hemorrhagic PED and hemorrhagic RD are at higher risk for breakthrough VH. Breakthrough VH secondary to PCV in different pachychoroid status has different clinical features and outcomes. Pachychoroid PCV patients are younger and associated with higher incidence of hemorrhagic complications. Pars plana vitrectomy for treatment of breakthrough VH in PCV patients is more complicated in pachychoroid PCV patients due to the higher incidence of hemorrhagic complications. In contrast, non-pachychoroid PCV patients are older and have more fibrovascular PED, similar to AMD. Despite that VH has developed, BCVA and hemorrhagic complications tend to improve significantly after adequate treatment. Higher frequency of anti-VEGF treatment and previous photodynamic therapy are associated with better visual prognosis in patients with breakthrough VH secondary to PCV.

## Supporting information

S1 Data (XLSX)
